# Oral Care Knowledge, Attitudes, and Practices of Black/African American Caregivers of Autistic Children and Non-Autistic Children

**DOI:** 10.3390/children9091417

**Published:** 2022-09-19

**Authors:** Dominique H. Como, Lucía I. Floríndez-Cox, Leah I. Stein Duker, Jose C. Polido, Brandi P. Jones, Mary Lawlor, Sharon A. Cermak

**Affiliations:** 1 Mrs. T.H. Chan Division of Occupational Science and Occupational Therapy, Herman Ostrow School of Dentistry, University of Southern California, Los Angeles, CA 90089, USA; 2 Nursing Research and Performance Improvement Department, Cedars-Sinai Medical Center, Los Angeles, CA 90048, USA; 3 USC Herman Ostrow School of Dentistry, Children’s Hospital Los Angeles, Los Angeles, CA 90027, USA; 4 Division of Dentistry, Children’s Hospital Los Angeles, Los Angeles, CA 90027, USA; 5 USC Race & Equity Center, University of Southern California, Los Angeles, CA 90089, USA; 6 Rossier School of Education, University of Southern California, Los Angeles, CA 90089, USA

**Keywords:** autism, African Americans, oral health, health equity, children

## Abstract

Oral health is a vital component of overall health. Children from underserved, minoritized populations (i.e., Black/African Americans, autistic children) are at even greater risk for experiencing oral health disparities. This study aims to illuminate the oral health knowledge, attitudes, and practices of Black/African American caregivers of autistic and non-autistic children. Black/African American caregivers of children (4-to-14 years) on the autism spectrum (*n* = 65) or not on the autism spectrum (*n* = 60), participated in a survey, with input from literature reviews, interviews, previous research, and reviews by experts. Caregivers demonstrated basic knowledge of oral health with significantly lower scores for caregivers of autistic children. Caregivers care about oral health and would like to increase their knowledge. Significant differences in oral care practices were found between the autistic and non-autistic groups. Caregivers reported they can access dental services with relative ease, including finding their child a dentist, scheduling a dental appointment, and accessing transportation (personal or public) to attend the visit. Black/African American caregivers of autistic children and children without autism seem to have foundational knowledge about oral health and basic practices; however, they are interested in learning more. Therefore, tailored oral health education programs may help mitigate oral health disparities for Black/African American families.

## 1. Introduction

Oral health is a vital component of overall health, impacting the quality of life across multiple daily activities, from one’s ability to confidently express emotions and engage with others to the completion of fundamental oral functions, such as chewing and swallowing [1]. Unfortunately, oral health is one of the most common chronic unmet health needs in the United States for both children and adults, with many families challenged by or unable to take advantage of preventive services [2,3,4,5]. Oral health has been a collective health priority in the nation following its inclusion in the Surgeon General’s report in 2000 [5]. Targeting oral health is critical to improving overall health, as poor oral health has been linked to heart disease, stroke, and diabetic complications, which overwhelmingly impact Black/African Americans in the United States [5]. Children from underserved minoritized populations, such as Black/African Americans, or those with special health care needs, including autistic children, are at even greater risk of experiencing oral health disparities [6,7,8,9,10]. This includes increased risk of caries (i.e., cavities or tooth decay) and oral-related chronic diseases, feeling more stigma and unequal treatment by their dental care provider, and poorer overall oral health status [5,11,12].

Despite significantly increased access to care and high rates of public insurance coverage among minoritized groups, Black/African American children continue to have higher rates of dental caries in their primary teeth (28%) and a significantly higher prevalence of untreated caries (nearly double) compared to Caucasian children [2,13]. Many factors serve as barriers to oral health for Black/African American children, including familial, social, and structural constructs, such as parental income and education level, race, disability status, insurance coverage, and the cost of services [6,14]. It has been suggested that Black/African American parents might have limited knowledge about risk factors and that cultural beliefs, attitudes, and norms may influence oral health practices, including in-home care and maintaining regular dental visits [15,16]. For example, oral care is affected by culture when there is a history of healthcare professional mistrust, a preference for cultural remedies, or belonging to an ethnic group with deeply held predispositions about preventive health [17,18,19].

Similarly, autistic children may experience barriers to oral health, including difficulty tolerating in-home and professional oral care practices, communication impairments, sensory processing differences, and uncooperative behaviors [10,20]. The prevalence rates of dental caries are inconsistent in autistic children (i.e., some studies show that autistic children have higher rates, while others have shown lower rates when compared to those without autism), however, seeking care has been a persistent problem [21]. For example, research shows that autistic children experience challenges brushing teeth, which may be due to their sensory processing differences and demonstrate uncooperative behaviors which can lead to the use of restraint or general anesthesia [9,10]. Additionally, some parents report that finding a dental practitioner willing and able to treat their child is difficult [22,23]. Furthermore, Black/African American autistic children may face unique and largely unresearched oral challenges. For example, Black/African American children are more likely to be diagnosed on the autism spectrum at a later age than their peers and often do not receive services in a timely manner [24,25]. This may delay their ability to engage in oral care preventative treatments (e.g., desensitization) or find an appropriate dental home, which may contribute to poor oral health outcomes. As the prevalence of autism continues to rise, it is increasingly likely that dental practitioners will encounter an autistic patient. Therefore, it is critical that practitioners, clinicians, and policymakers are aware of the needs of this population.

This study aims to illuminate the oral health knowledge, attitudes, and practices of Black/African American caregivers of autistic children and children not on the autism spectrum and identify areas where future research or intervention programs should be focused.

## 2. Materials and Methods

This data is a part of a larger convergent parallel mixed-method study designed to understand the barriers and facilitators to oral care for Black/African American families with autistic children and children not on the autism spectrum. This study was approved by the University of Southern California Institutional Review Board (HS-19-00995).

### 2.1. Participants

The total sample size of this study was 125 parents or primary caregivers (e.g., grandparents, guardians) [both parents and primary caregivers referred to as ‘caregiver(s)’ in this paper], with children aged 4 to 14 years, on the autism spectrum (*n* = 65) or not on the autism spectrum (*n* = 60). The sample size was determined based on published studies with similar topics [12]. Eligibility criteria for participants were met if they (a) self-identified as Black/African American or multiracial (including Black/African American) and identified their child similarly, (b) had a child aged 4–14 years, (c) read/understand English, and (d) were a resident of the United States. In addition, for inclusion in the autistic group, a child needed a diagnosis on the autism spectrum. Autism diagnosis was based on caregiver report and validated on the Social Responsiveness Scale, Second Edition (SRS-2) [26]. Those children with a T-score of 59 or below on the SRS-2 and described by their caregiver as not diagnosed on the autism spectrum were included in the non-autism group. Those with a T-score of 60 or above who were described by their caregiver as diagnosed on the autism spectrum were included in the autistic group. Participants were excluded if their child had a diagnosis which might severely impact oral health (e.g., Down syndrome, Cerebral Palsy).

### 2.2. Procedures

Caregivers were recruited via advertisements posted to social media (e.g., Facebook, Instagram, Reddit) and Research Match; a list of previously recruited study participants who agreed to be contacted about future research opportunities were also emailed study information for recruitment purposes. Respondents who indicated interest were provided with a link to an online survey. The online survey was hosted on Research Electronic Data Capture (REDCap), a secure web-based platform hosted at the University of Southern California, where the data was also stored. Prior to beginning the survey, respondents were provided with a brief written introduction to the purpose of the study, and informed that participation was voluntary and anonymous. Caregivers completed an approximately 20-min online survey, the Oral Health Questionnaire (OHQ), which was developed with particular attention to racial-ethnic minority groups (i.e., Black/African American and Latino/a/x) and autistic populations. In addition, participants completed the SRS-2 to confirm the diagnosis, or lack, of autism. All participants were offered a $15 gift card for completing these assessments. Data collection occurred from April 2020 to March 2021.

#### 2.2.1. Social Responsiveness Scale, Second Edition (SRS-2)

The SRS-2 is a 65-item tool used to identify the presence and severity of social impairments, which are often evident within the autism spectrum. The SRS-2 serves as a reliable clinical measure of autism [26]. The severity score is categorized into one of four classifications: severe (T-score of 76 and above), moderate (T-score of 66–75), mild (T-score of 60–65), within normal limits (generally not associated with clinically significant autism; T-score of 59 and below).

#### 2.2.2. Oral Health Questionnaire (OHQ)

The OHQ was developed (by authors Floríndez-Cox & Como) based on literature reviews of oral care, interviews with minoritized and medically underserved populations, and findings from previous research. We generated a list of possible items based on this review. These items were reviewed by experts in pediatric dentistry, nutrition, occupational therapy, public health, survey development (e.g., biostatistics, psychometrics), and minority health. Items were reviewed multiple times by reviewers with expertise in health promotion to assure that they reflected the category of knowledge, attitude, or practice. This questionnaire has previously been used to examine oral care in Latino/a/x populations [12]. The OHQ is a 120-item survey assessing oral health knowledge (21-items), attitudes (38-items), and practices (23-items), as well as access (8-items) and demographic questions. The topics covered relate to caregiver oral health knowledge (i.e., causes of cavity formation), caregiver and child in-home oral health practices (i.e., frequency and duration of toothbrushing), dental and oral health attitudes (i.e., motivation for going to the dentist, keeping teeth healthy), access to dental care/treatment (i.e., availability of dental services), and demographics about the caregiver and characteristics of the child. In addition, questions were asked about oral care education (i.e., willingness and preferred method to receive additional information about oral care).

The knowledge questions consisted of ‘True-False-I don’t know’ answer responses, which were scored as correct or incorrect, with an “I don’t know” response treated as a wrong answer. Questions about attitude used a 7-point Likert scale, where caregivers were invited to express their level of agreement with a statement. The scores were converted to a numerical value of 1 to 7 (agree responses 5–7; disagree responses 1–4). The oral care practice questions asked about caregiver and child oral care activities. Items were scored with either a (0), (1), or (2) based on the question. Frequency questions (i.e., “how often do you usually brush your teeth?”) with a 0-score being given for a response of “never”, a score of (1) for a reply equivalent to “sometimes/partial”, and a 2-score for meeting or exceeding established professional recommendations. “Yes/No” questions (i.e., “does your child floss?”) also utilized a 0–2 scale with a “no” response obtaining a (0), “sometimes” a (1), and a “yes” a (2). The scores for each item were summed to obtain the section score, with a higher score indicating more health-facilitating behaviors. Questions were reverse-scored if they asked about negative attitudes, access beliefs, or practices. Access to care and demographic questions were descriptive in nature. This survey is available from the first author upon request.

### 2.3. Analysis

Data from the survey was imported into a statistical analysis software package (IBM SPSS Statistics for Windows, Version 27.0, Armonk, NY: IBM Corp) for analysis. Descriptive statistics were conducted for demographics, level of oral care knowledge, reported frequency of oral care practices, and attitudes toward oral health. For descriptive purposes, means, standard deviations, frequencies, and percentages were calculated for each survey variable and demographic variables. *T*-tests, chi-squares, and Fisher’s exact tests were utilized to document differences between autistic and non-autistic groups, as appropriate. Normal distribution was determined using Kolmogorov-Smirnov and Shapiro-Wilk test of normality in SPSS. Pearson or Spearman’s rho correlations (based on data normality) were used to assess the relationship between demographic variables, knowledge, attitudes, practices, and access to care as appropriate. A two-sided 0.05 level of significance was utilized.

## 3. Results

### 3.1. Demographic Characteristics

The demographic characteristics of the study participants are shown in Table 1.

Of the 125 participants, most self-identified as Black/African American mothers/stepmothers who were currently employed and possessed dental insurance for themselves and their child. There was a significant difference in race *t*(123) = 4.994, *p* ≤ 0.001 between the autistic and non-autistic groups, with the non-autistic group having 19 (31.7%) participants identifying as multiracial compared to 10 in the autistic group (1.5%). Significant differences were also noted in education *t*(123) = 3.958, *p* ≤ 0.001; income *t*(123) = 2.556, *p* ≤ 0.012; and marital status *t*(123) = −5.179, *p* ≤ 0.001 between the autistic and non-autistic groups, with the non-autistic group reporting more years of education completed and higher incomes, while the autistic group reported higher rates of marriage/domestic partnership (89.2% autistic; 50% non-autistic).

### 3.2. SRS-2

The SRS-2 was administered to all caregivers. Scores of 60 participants were within normal limits, aligning with the caregiver’s report of no autism diagnosis (non-autistic group). Of the 65 children in the autistic group, the majority (66%) fell into the moderate severity level (see Table 1). Scores in this range indicate deficiencies in reciprocal social behavior that may substantially interfere with everyday interactions.

### 3.3. Oral Health Questionnaire (OHQ)

#### 3.3.1. Oral Health Knowledge (Knowledge)

The knowledge section consisted of 21 questions. The average number of questions correctly answered for the whole sample was 14.06 (*SD* ± 4.2), with a significantly lower score for caregivers of autistic children (*M* = 12.68 ± 3.6) compared to those in the non-autistic group (*M* = 15.55 ± 4.4), *t*(123) ≤ 3.999, *p* < 0.001. The questions that less than half of all respondents answered correctly concerned the relationship of poor oral health to other chronic conditions (i.e., stroke, lung cancer). There was a significant difference in correct responses from caregivers between the two groups (autistic and non-autistic) on 10 of the 21 knowledge items. A higher percentage of caregivers in the non-autistic group were correct in 8 out of 10 of these items. The items included topics regarding the cause and prevention of bacteria (plaque) and cavities (e.g., toothpaste with fluoride helps to prevent cavities, flossing daily helps prevent plaque from forming between teeth, plaque causes cavities) and the impact of sugary foods on oral health (e.g., limiting sugary snacks helps prevent cavities, carbohydrates can break down into sugar and can harm teeth), with more caregivers of autistic children responding incorrectly. See Table 2 for the significant differences found in the OHQ sections for families with autistic children and non-autistic children.

#### 3.3.2. Dental and Oral Health Attitudes [Attitudes]

Overall, most respondents (79.2%) indicated that they care about oral health as much as general health. Caregivers (89.6%) reported that they trust their child’s dentist and believe their child receives high-quality care from their dentist (86.4%). Those who indicated they did not trust their child’s dentist were all from the non-autistic group (10.4%). Just about half of the caregivers reported for themselves (51.2%) and their child (50.4%) that they were fearful of going to the dentist. More than half (52%) of the caregivers believe their race/ethnicity negatively influences their child’s treatment. The majority (58.4%) reported they agree (i.e., somewhat, strongly) that they would prefer their child’s dentist to be of the same race/ethnicity, with more of the caregivers of the autistic group in agreement. Nearly all participants (94.4%) agreed they would be willing to take time off from work to get their child to a dental visit, although most caregivers (68.8%) also agreed that they wished their dentist had more flexibility in their appointment availability. Most participants (80%) reported prioritizing their child’s dental health over their own. The autistic group was asked if they believe that their child’s autism makes oral care activities challenging to complete, and most caregivers (86.2%) reported in the affirmative.

Significant differences in attitudes between caregivers of autistic children and non-autistic children were noted regarding their ability to prevent cavities, aid their child to have healthy teeth, the eminent loss of teeth in old age, and the importance of other health problems over dental disease, with caregivers of the autistic group indicating more agreement with the statements, denoting negative attitudes about self-efficacy to prevent oral health issues (Table 3).

There were also significant differences between the autistic and non-autistic groups regarding food choices and preparation. While most caregivers agree that cooking meals at home allows for better monitoring of their child’s sugar intake, nearly all the caregivers of autistic children agreed (98.5%) compared to caregivers of the non-autistic group (78.3%). Furthermore, a significant difference was noted in caregivers’ ability to get their child to eat foods that promote healthy teeth. More caregivers of autistic children (81.5%) agreed it was easy compared to the non-autistic group (51.7%). Most caregivers of autistic children (93.8%) indicated that their child’s food preferences make eating non-sugary foods difficult.

#### 3.3.3. Caregiver and Child In-Home Oral Care Health Practices [Practices]

Slightly more than half of all caregivers reported that, on average, their child has been to the dentist once (28.8%) or twice (25.6%) in the last year, while over 40% reported that their child had not been to the dentist in the previous year. However, most caregivers (60%) said they had not had a dental cleaning in the last year; 20.8% reported one cleaning, and 17.6% reported two cleanings. Moreover, 4% of the children and 1.6% of the caregivers went to the dentist for a cleaning three times last year. In the home, just over half of the caregivers reported that their child brushes their teeth at least twice per day (59%) for at least 120 s (26.4%) each time and usually flosses (65.6%). For themselves, 62.4% of caregivers reported brushing their teeth twice per day or more for 120 s or more (34.4%) and regularly flossing during oral care (56.0%).

Significant differences between the autistic and non-autistic groups were found in 5 of the 21 oral care practice items. Caregivers of the autistic group (87%) reported their child usually flosses (with or without assistance), while only 41.7% of non-autistic group caregivers reported their child usually flosses. This difference also extended to the caregivers, as the caregivers in the autistic group usually floss when they brush (81.5%) versus less than 30% of the non-autistic group. Significant differences were also noted in the number of minutes spent brushing their teeth, with the non-autistic caregivers and child brushing for longer than those in the autistic group (see Table 4 for values).

#### 3.3.4. Access to Dental Care/Treatment (Access)

The majority of all respondents reported that they agreed (i.e., somewhat, strongly) that they can access dental services for their child with relative ease, including finding their child a dentist (70.4%), scheduling a dental appointment (71.2%), and accessing transportation (personal or public) (91.2%). Caregivers also agreed that their child has a regular dentist (91.2%), dental care for their child is affordable (74.4%), and their child’s dentist has business hours that work with their schedule (73.6%). Significant differences were noted between the responses to the access questions in the autistic and non-autistic groups. The caregivers in the autistic group seemingly had more perceived access. Caregivers of the autistic group more strongly agreed with statements regarding access to affordable dental care (*p* ≤ 0.001), obtaining dental insurance for their child (*p* ≤ 0.001), finding a dentist (*p* ≤ 0.002), and scheduling appointments with ease (*p* ≤ 0.003) compared to the caregivers of the non-autistic group. A significant difference was also noted in non-autistic caregivers who report they are largely unable to find a dentist of the same race/ethnicity as them (*p* ≤ 0.001) compared to the autistic group.

### 3.4. Relationships between Variables of Oral Care

Correlations were computed to assess relationships between knowledge, practices, attitudes, access, and specific demographics (e.g., education, income). There was a small positive significant correlation between caregiver knowledge and the family’s oral care practices, *r*(123) = 0.211, *p* ≤ 0.018, and with knowledge and attitudes, *r*(123) = 0.686, *p* < 0.001.

There was no significant relationship between caregiver knowledge and their perceived access to services. Nor was there a significant relationship found between caregiver perceived access to oral care services, their oral care practices, and caregiver attitudes. However, a significant positive correlation was found between oral care practices and attitudes, *r*(123) = 0.377, *p* ≤ 0.001. There were also significant positive correlations for the following scales and demographics: knowledge and income, *r*(123) = 0.215, *p* ≤ 0.016; knowledge and education, *r*(123) = 0.507, *p* ≤ 0.001; attitudes and education, *r*(123) = 0.459, *p* ≤ 0.001; and attitudes and income, *r*(123) = 0.199, *p* ≤ 0.026. As expected, income and education were significantly related.

There were significant relationships between oral health knowledge, access, attitudes, and practices for each of the groups. For the autistic group, there was a positive correlation found between attitudes and practice, *r*(60) = 0.483, *p* ≤ 0.001; knowledge and attitudes, *r*(60) = 0.480, *p* < 0.001; knowledge and practices, *r*(60) = 0.308, *p* ≤ 0.013; and a negative correlation between attitudes and access, *r*(60) = −0.287, *p* ≤ 0.020; and knowledge and access, *r*(60) = −0.273, *p* ≤ 0.028. For the non-autistic group, there were positive correlations between access and knowledge, *r*(55) = 0.537, *p* ≤ 0.00; attitudes and access, *r*(55) = 0.650, *p* ≤ 0.001; attitudes and practice, *r*(55) = 0.373, *p* ≤ 0.003; and attitudes and knowledge, *r*(55) = 0.744, *p* ≤ 0.001.

In summary, caregivers in both groups who reported more favorable oral health attitudes also demonstrated better oral health practices and more oral health knowledge. For the non-autistic group, oral health attitudes and knowledge improved as perceived access to oral health services increased. Similarly for the autistic group, oral care practices increased as oral health knowledge increased; conversely, when access decreased, attitudes and knowledge about oral health increased.

### 3.5. Ongoing Needs

Most of the respondents (80%) reported that they would like to increase their oral health knowledge and indicated that they would like to learn more ways to improve their child’s oral health (88%). Furthermore, 60% of respondents reported previously trying to find information to enhance their child’s oral health; of these, the most frequent sources included health professionals (31.2%), the internet (27.2%), and friends or family (13.6%). The majority of the caregivers (68.0%) reported that they would be at least somewhat likely to participate in a free education program. The preferred method of delivery was online (64.8%), followed by a paper method (e.g., pamphlet) (30.4%), and one percent of the respondents would prefer an in-person method. At the same time, the remaining caregivers indicated “no preference”. That said, the majority of the caregivers (71.2%) indicated they would be at least somewhat likely to participate in a community oral health initiative (i.e., oral exams, dental cleanings) that occurred at their child’s school. There were no significant differences between the autistic and non-autistic groups.

## 4. Discussion

The findings of this study highlight the relationships between knowledge, attitudes, practices, access, and various demographics that impact oral health for Black/African American families of autistic children and children without autism. This study draws attention to the gaps, behavior patterns, and cultural beliefs largely unrecognized in oral health for these intersecting identities, with differences noted between caregivers of autistic children and caregivers of non-autistic children.

Limited knowledge has been suggested as one possible reason for the persistence of oral health disparities in Black/African American caregivers [15]. One of the main areas in which oral health knowledge was limited for all caregivers was the relation of poor oral health to other chronic conditions (i.e., heart failure, stroke, diabetes, lung cancer), which disproportionally impact Black/African Americans [5]. Black/African Americans’ health is often affected by increased risk factors and higher incidence, morbidity, and mortality rates of many chronic diseases and conditions compared with White Americans [27]. African Americans had 40% higher rates of all-cause deaths in all age groups less than 65 years for heart disease, stroke, and diabetes than Caucasians [28].

The caregivers of the autistic group also showed decreased knowledge about the negative impact of sugary foods (e.g., carbohydrates, desserts) on oral health outcomes. Sweet snacks and foods such as cereal, french fries, and potato chips break down to sugar in the mouth and can harm teeth, frequently leading to higher caries rates in children [29]. African American children are at increased risk for developing early childhood caries based on food consumption [30]. Caregivers in this study reported that getting their child to eat health-promoting foods was easy while also stating that their child’s food preferences make it difficult to eat non-sugary foods. These responses seem incongruent with one another and deserve further attention. Despite this, evidence suggests that limiting the consumption of these snacks can help prevent cavities. Therefore, it is essential that caregivers understand how sugar affects teeth so they can make food choices for their families that support good oral health.

Black/African American caregivers overwhelmingly reported seeking additional opportunities to obtain knowledge to improve their child’s oral health. As is evident in this study, increased dental knowledge has been shown to have a positive correlational relationship with more positive oral health attitudes and practices. Thus, a tailored oral health education program that focuses on the aforementioned topics may be an opportunity to mitigate oral health disparities for Black/African American families. For example, caregivers may benefit from an educational program that: (1) highlights the connection between oral health and overall health, specifically its impact on chronic conditions; (2) demonstrates how sugar can lead to increased caries while also considering the limited food repertoire some autistic children may have; and (3) provides resources that can be easily accessed by caregivers. A similar self-care educational program has been successfully implemented for Black/African American adolescents [31]. Community-based educational programs may have an even larger impact as one study found that Black/African Americans caregivers were more likely to obtain their oral health information from those in their community (i.e., teachers, babysitter, support group) [32].

One of the leading strategies for reducing oral health disparities in the United States has been to increase access to dental services through insurance and other low-cost discount plans. For instance, the funding of preventive services has increased for Medicaid coverage plans, and dental benefits must be included by any state that provides Children’s Health Insurance Program (CHIP) coverage through a Medicaid expansion program [3]. Evidence suggests that a direct outcome is that access to care has significantly increased and that the rates of public insurance coverage are most significant among minorities [3]. This holds true here, as the majority of caregivers report having dental insurance for themselves and their child. As a result, they also say dental care is largely affordable, likely due to higher insurance coverage rates. While accessing dental services was a largely positive experience for many caregivers, finding a dentist for those without autism was surprisingly more challenging than for those on the autism spectrum. This is contrary to literature which indicates that caregivers of autistic children report that finding a dentist for their child is difficult [22,33]. It is possible that the respondents of this survey were mainly from urban areas where specialized services were more readily available. It is also possible that caregivers of autistic children might have strong networks, whether collegial or professional, who utilized word-of-mouth referrals to find dental practitioners willing and able to provide services to their child.

Despite indications from caregivers that dental services were accessible with high insurance coverage rates, there was still a large portion of the caregiver participants who, in the previous 12-month period, had not been to the dentist or had not taken their child to the dentist. It is possible that this was a result of the sampling period, which included a time when COVID-19 pandemic precautions were being implemented across the country with various restrictions. Conversely, in-home oral care practices, such as brushing and flossing, occurred daily for the caregiver and child in autistic and non-autistic groups. This finding is similar to the results of study that examined the toothbrushing and oral care habits of Latino/a/x autistic and non-autistic children [10].

Some of the most interesting findings related to caregiver attitudes about their child’s dentist. Positive attitudes were related to better oral care practices, whether in the home or at the dental clinic. While most caregivers trusted their child’s dentist, those who did not were from the non-autistic group. A possible explanation may reflect the differences in how the two groups find a dentist (e.g., independent search vs. word of mouth, referrals). Another explanation may be the type of dentist they seek services from. For example, dentists who work with children with special health care needs may receive additional training, resulting in a more favorable experience. It is also possible that caregivers of children with special health care needs are more familiar with the concept of medical homes and seek consistency in their health teams.

Additionally, nearly half of the respondents reported that they are fearful of dentists and say the same for their child, which is much larger than that reported in the larger population, where dental anxiety is reported to be around 15% in adults and range from 5% to 20% for children [34,35,36]. This is an essential factor to consider as caregivers are primarily responsible for introducing and implementing good oral health practices to their child and may inadvertently transfer their fear and anxiety. In addition, this may influence their general attitudes about dentists. High dental fear and anxiety rates were also found in a Latino/a/x population [12]. The higher rates for minoritized groups are worth exploring to determine what has led to these increased rates.

Caregivers indicated that patient-provider relationships might be strained as more than half stated that they felt their family’s race/ethnicity negatively influences how their child is treated at the dentist. Homophily, the tendency to bond with similar people, may be a potential facilitator for overcoming patient-provider mistrust and oral health disparities. More than half of the participants reported that they would prefer for their child’s dentist to be of the same race/ethnicity as themselves (80% autistic group; 35% non-autistic group), which may be linked to their belief that race/ethnicity negatively influences how their child is treated. However, this is not easily achieved for Black/African American patients and practitioners and may be attributed to the disproportionate growth of minoritized populations compared to dentists with minority backgrounds. According to a report produced by the American Dental Association, African Americans comprise 12.4% of the population, while only 3.8% of dentists self-identified as African American [37].

Caregiver education level and income are known social determinants for children’s overall health. This extends to oral health as well. Lower caregiver education has been associated with increased dental caries; higher caregiver education was associated with an increased likelihood of using dental services yearly for their child [38,39]. This research gives support to the impact that social determinants of health (i.e., caregiver education and income) have on oral health as knowledge was positively correlated with education, and attitudes were positively correlated with income and education. This is important because the autistic group had lower education and income levels and negative attitudes about self-efficacy to prevent oral health issues, possibly contributing to persisting oral health disparities.

### Limitations

This work has many strengths, including the coverage of a broad range of health factors (i.e., knowledge, attitudes, practices, access, culture) on oral health outcomes in a minoritized population. The survey represented a critical integration of culturally based concepts into a questionnaire. The moderate sample size of this study may be viewed as a limitation; however, recently published studies exploring oral care in minoritized populations had smaller samples [12,40]. As this is an emergent field of study, it follows that sample sizes will increase as more research is conducted. Another possible limitation is the fact that most survey respondents identified as mother/stepmother, which may impact the data. Additionally, as we did not collect identifying information from respondents, we have no way to ensure that caregivers only completed the survey once. However, due diligence and a thorough review were utilized to minimize multiple responses (i.e., IP address review). Finally, although the survey was developed based on the literature and expertise of individuals from different disciplines, its psychometric properties were not evaluated.

## 5. Conclusions

Black/African American caregivers of autistic children and children without autism seem to have foundational knowledge about oral health and basic practices; however, they are interested in learning more. Thus, tailored oral health education programs may be an opportunity to mitigate oral health disparities for Black/African American families. Most families expressed a preference for having the education provided online. This likely reflects the difficulty with transportation, time off work, and finding appropriate childcare. Perhaps COVID-19 has made online education a more acceptable method of receiving information. Patient-provider relationships may need to be strengthened as many families indicated they experienced dental fear and felt their race/ethnicity negatively impacted how the dentist treated them. Creating culturally tailored educational and behavioral interventions may help to improve oral health disparities for Black/African American families. It is essential to continue to explore the intersection of culture and oral health for this population.

## Figures and Tables

**Table 1:** Demographics.

	Total	Autistic	Non-Autistic
*n* =	125 *n* (%)	65 *n* (%)	60 *n* (%)
Questionnaire completed by:			
Mother/Stepmother	95 (76%)	42 (64.6%)	53 (88.3%)
Father/Stepfather	25 (20%)	22 (33.8%)	3 (5%)
Guardian	5 (4%)	1 (1.5%)	4 (6.7%)
Caregiver age	37.45 (*SD* ± 7.2)	37.05 (*SD* ± 7.0)	37.88 (*SD* ± 7.4)
Cargiver race/ethnicity			
Black	105 (84%)	64 (98.5%)	41 (68.3%)
Multiracial (including Black)	20 (16%)	1 (1.5%)	19 (31.7%)
Child gender			
Male	66 (52.8%)	33 (50.8%)	33 (55%)
Female	59 (47.2%)	32 (49.2%)	27 (45%)
Child age	8.94 (*SD* ± 3.0)	9.02 (*SD* ± 2.6)	8.87 (*SD* ± 3.4)
Child race/ethnicity			
Black	104 (83.2%)	63 (96.9%)	41 (68.3%)
Multiracial (including Black)	21 (16.8%)	2 (3.1%)	19 (31.7%)
Autism severity (SRS-2)			
Not Present	60 (48%)	-	60 (100%)
Mild	3 (2.4%)	3 (5%)	-
Moderate	43 (34.4%)	43 (66%)	-
Severe	19 (15.2%)	19 (29%)	-
Child overall health			
Excellent	16 (12.8%)	3 (4.6%)	13 (21.7%)
Very good	44 (35.2)	22 (33.8%)	22 (36.7%)
Good	50 (40.0)	31 (47.7%)	19 (31.7%)
Fair	15 (12.0)	9 (13.8%)	6 (10%)
Poor	0	0	0
Caregiver overall health			
Excellent	9 (7.2%)	3 (4.6%)	6 (10%)
Very good	32 (25.6)	16 (24.6%)	16 (26.7%)
Good	51 (40.8)	27 (41.5%)	24 (40%)
Fair	27 (21.6)	15 (23.1%)	12 (20%)
Poor	6 (4.8)	4 (6.2%)	2 (3.3%)
Dental insurance			
Caregiver			
Yes	104 (83.2%)	55 (84.6%)	49 (81.7%)
No	21 (16.8%)	10 (15.4%)	11 (18.3%)
Child			
Yes	118 (94.4%)	59 (90.8%)	59 (98.3%)
No	7 (5.6%)	6 (9.2%)	1 (1.7%)
Siblings			
Yes	99 (79.2%)	54 (83.1%)	45 (75%)
No	26 (20.8%)	11 (16.9%)	15 (25%)
Education			
<High School	4 (3.2%)	0	4 (6.7%)
Some HS/Completed HS	23 (18.4%)	13 (20%)	10 (16.7%)
Some college/Associate’s degree	61 (48.8%)	49 (75.4%)	12 (20%)
Bachelor’s degree	16 (12.8%)	1 (1.5%)	15 (25%)
Graduate/Post Graduate degree	21 (16.8%)	2 (3.1%)	19 (31.7%)
Marital Status			
Married/Domestic Partnership	88 (70.4%)	58 (89.2%)	30 (50%)
Single/Divorced/Widowed	37 (29.6%)	7 (10.8%)	30 (50%)
Household income			
<20,000	12 (9.6%)	5 (7.7%)	7 (11.7%)
20,000–34,999	11 (8.8%)	1 (1.5%)	10 (16.7%)
35,000–49,999	20 (16.0%)	7 (10.8%)	13 (21.7%)
50,000–74,999	51 (40.8%)	42 (64.6%)	9 (15.0%)
75,000–99,999	17 (13.6%)	8 (12.3%)	9 (15.0%)
100,000+	14 (11.2%)	2 (3.1%)	12 (20%)
Employment Status			
Employed	101 (80.8%)	51 (78.5%)	50 (83.3%)
Unemployed	24 (19.2%)	14 (21.5%)	10 (16.7%)

**Table 2:** Significant Differences in Individual Items of Oral Care Knowledge of Families with Autistic Children And Children Without Autism.

Knowledge	Autistic *n* = 65 *n* (% Correct)	Non-Autistic *n* = 60 *n* (% Correct)	*p*
Children don’t need their own toothbrush until all their teeth come in.	60 (92.3%)	48 (80.0%)	0.045
Using toothpaste with fluoride helps prevent cavities.	36 (55.4%)	47 (78.3%)	0.007
Flossing daily helps prevent bacteria (plaque) from forming between teeth.	35 (53.8%)	56 (93.3%)	<0.001
Cavities are caused by bacteria (plaque) that forms on teeth.	38 (58.5%)	53 (88.3%)	<0.001
Limiting sugary snacks (desserts, chocolate, candies) helps prevent dental cavities.	26 (40.0%)	56 (93.3%)	<0.001
Drinking fruit juice is better than eating candy for your teeth.	47 (72.3%)	32 (53.3%)	0.028
Foods containing carbohydrates (such as bread tortillas, pizza crust, cereal, fries, potato chips) break down into sugar in the mouth and can harm teeth.	25 (38.5%)	45 (75.0%)	<0.001
Dental problems are related to heart failure.	18 (27.7%)	44 (73.3%)	<0.001
Dental problems are related to stroke.	12 (18.5%)	23 (38.3%)	0.013
Dental problems are related to diabetic complications.	16 (24.6%)	35 (58.3%)	<0.001

Note: Chi-Square Test.

**Table 3:** Significant Differences in Individual Items of Oral Care Attitudes of Families with Autistic Children and Children Without Autism.

Attitudes	Autistic *n* = 65 *n* (% agree)	Non-Autistic *n* = 60 *n* (% agree)	*p*
I trust my child’s dentist.	65 (100%)	47 (78.3%)	<0.001
My family cares about oral health as much as general health.	59 (90.8%)	40 (66.7%)	0.002
Meals I cook at home allow me to better monitor my child’s sugar intake than meals at restaurants.	64 (98.5%)	47 (78.3%)	<0.001
Getting my child to eat foods (carrots, leafy greens, etc.) that are good for promoting healthy teeth is easy.	53 (81.5%)	31 (51.7%)	<0.001
My child’s food preferences make eating non-sugary foods difficult.	61 (93.8%)	33 (55%)	<0.001
I prefer that my child’s dentist be the same race/ethnicity as me.	52 (80%)	21 (35%)	<0.001
I think my family’s race/ethnicity negatively influences how my child is treated at the dentist.	48 (73.8%)	17 (28.3%)	<0.001
There is nothing I can do to prevent cavities in my child.	50 (76.9%)	6 (10%)	<0.001
There is not much I can do to help my child have healthy teeth.	43 (66.2%)	5 (8.3%)	<0.001
Dental disease is less important than other health problems.	49 (75.4%)	18 (30%)	<0.001
It is natural for people to lose their teeth in old age.	55 (84.6%)	34 (56.7%)	<0.001
I prioritize my child’s dental health over my own dental health.	59 (90.8%)	41 (68.3%)	0.003
I am fearful of going to the dentist.	40 (61.5%)	24 (40%)	0.020

Note: Fisher’s Exact Test.

**Table 4:** Significant Differences in Individual Items of Oral Care Practices Families with Autistic Children and Children Without Autism.

Practices	Autistic *n* = 65 *	Non-Autistic *n* = 60 *	*p*
* see measure in parenthesis next to each question			
I reward my child with candy, soda, chocolate, ice cream or other sugary treats. * *n* (% yes)	10 (15.4%)	23 (38.3%)	0.004
How many minutes does your child spend brushing his/her teeth? * (Average seconds (secs))	<30 s	60–90 s	<0.001
On average, how many minutes do you spend brushing your teeth? * (Average seconds (secs))	60–90 s	≥120 s	0.002
Does your child floss (with or without assistance/prompts)? * *n* (% yes)	57 (87.7%)	25 (41.7%)	<0.001
Do you floss when you brush your teeth? * *n* (% yes)	53 (81.5%)	17 (28.3%)	<0.001

Note: Chi-Square Test.

## Data Availability

For more information on the sample or for inquiries about use of the OHQ, please contact the first author.
